# Enhancing student performance with multicolored 3D printed neuroanatomical models in veterinary education

**DOI:** 10.1186/s12909-025-07908-y

**Published:** 2025-10-02

**Authors:** Nedžad Hadžiomerović, Rizah Avdić, Adis J. Muminović, Abdullah Muftić, Adi Pandžić, Faruk Tandir, Anel Vejzović, Ozan Gündemir, Amina Isanović Hadžiomerović

**Affiliations:** 1 Department of Basic Sciences of Veterinary Medicine, University of Sarajevo – Veterinary Faculty, Sarajevo, Bosnia and Herzegovina; 2 Department of Mechanical Design, Faculty of Engineering, University of Sarajevo, Sarajevo, Bosnia and Herzegovina; 3 Department of Pathobiology and Epidemiology, University of Sarajevo – Veterinary Faculty, Sarajevo, Bosnia and Herzegovina; 4 Department of Mechanical Production Engineering, Faculty of Engineering, University of Sarajevo, Sarajevo, Bosnia and Herzegovina; 5 Department of Anatomy, Faculty of Veterinary Medicine, Istanbul University- Cerrahpasa, Istanbul, Türkiye; 6 Department of Education, Faculty of Philosophy, University of Sarajevo, Sarajevo, Bosnia and Herzegovina

**Keywords:** 3D printing, Models, Neuroanatomy, Veterinary education, Students’ satisfaction

## Abstract

**Background:**

The neuroanatomy course consistently presents significant challenges, primarily due to the short lifespan of formalin-preserved brain specimens and their restricted availability. Numerous studies have evaluated the strengths and limitations of alternative resources for neuroanatomy education, with a particular focus on technology-based learning methods. This study aimed to assess the effectiveness of integrating 3D printed models into the neuroanatomy curriculum and to evaluate student satisfaction with their use.

**Methods:**

The experimental group consisted of the first-year students enrolled in the integrated study program at the University of Sarajevo-Veterinary Faculty during the summer semester of the academic year 2022/2023. The course was organized using 3DP models for 15 h, while during the remaining three hours formalin-preserved specimens were utilized. Data obtained from this cohort were compared with the results of the previous two cohorts (2021–2022 and 2020–2021), who studied veterinary anatomy exclusively using formalin-preserved specimens.

**Results:**

The neuroanatomy test scores improved in the experimental group compared to the two control groups. The students exhibit positive attitudes and report high satisfaction with using 3DP models. They support innovative teaching methods and find that the colored segments of the models enhance recognizability of different anatomical structures, highlighting the didactical value of 3DP models.

**Conclusion:**

Overall, study demonstrated that 3DP models were highly beneficial for neuroanatomy learning, pedagogically useful and well-received by students.

## Introduction

Anatomy is a cornerstone of medical and veterinary education with a strong scientific and clinical significance. Knowledge of anatomy is essential for understanding physiology, pathology and clinical work providing a platform for all medical professions [1, 2]. The anatomy education went through remarkable changes evolving from traditional cadaver dissection which is considered as the “gold standard” of anatomical education to the new technology enhanced learning solutions [3, 4]. Cadaver-based instruction became challenging due to reduced teaching hours dedicated to this content in the curricula and the difficulties associated with maintaining anatomical laboratories [5, 6]. Additional challenges involve ethical concerns about using cadavers and the hazardous chemicals required for tissue preservation [7–9]. To address most of these challenges, new instructional tools must be non-toxic, cost-effective, accessible, and user-friendly, allowing students to engage in interactive learning both inside and outside anatomy laboratories [10, 11]. The COVID-19 pandemic prompted educators in all fields of medicine to innovate their approaches to incorporate more flexible and digital teaching methods [12]. Educators were compelled to swiftly transition to distance education and utilize digital teaching resources [13]. Resources such as virtual reality (VR), augmented reality (AR), three-dimensional (3D) models, video lectures, podcasts, 3D printed models become the “new age” of anatomy teaching [14, 15].

3D printing or additive manufacturing is a technique for creating physical objects or models from various digital data sets. Over the past 10 years, 3D printing has become increasingly popular in the medical field and anatomy education [16, 17]. The notable rise in the use of 3D printing (3DP) technology is linked to the broad range of methods for acquiring 3D models, including CT and MRI scans, 3D modeling, and 3D scanning technology [18]. The 3D printing technology went from a quite costly to relatively affordable tool in the last decade [19]. In veterinary medicine, 3D printing is utilized to create precise personalized anatomical models for education, training, and research. It is also used to produce customized surgical implants and prosthetics, as well as to treat injuries to animal Limbs, fins, beaks, bones, and shells. Additionally, 3D printing is used for tissue-engineered scaffolds, tailored surgical instruments, canine masks, and orthopedic applications [2, 20]. The two widely applied 3D printing methods are fused deposition modelling (FDM), which involves melting filament, and stereolithography (SLA) based on polymerization and curing resin [21]. It is reported that FDM technology has been used to create a portable horse head model for training in effective and humane equine gunshot euthanasia and to produce 3D models of nonunion fractures in long bones as educational tools [22, 23].

There are already studies evaluating educational outcomes of the 3D printed models in anatomy teaching. Lim et el. found the significant improvement in the students’ test scores of the 3D printed group compared to the cadaveric or combined materials groups [24]. In another study, the use of various 3DP models resulted in a significant increase in students’ scores compared to the 2D image-based models [4]. Moreover, 3D printed models enhanced learning of neuroanatomical content and understanding of the three-dimensional relations of anatomical structures. The high-quality printed models and adequate specimen-student ratio during self-directed learning session resulted in higher scores compared to the formalin-preserved specimens [25]. However, several studies revealed that students preferred plastinated specimens over 3DP models and showed similar performance outcomes [26, 27].

The use of technology in medical education is constantly evolving, especially in the post-COVID era. Advanced imaging techniques like X-rays, CT scans, MRI provide information about internal organs, while recent advancements in 3D scanning technology enable detailed data collection from outer body parts. The use of 3D scanning in medical field includes the creation of accurate prostheses, various implants, anatomical and surgical models, preoperative planning, and developments in the dental sector [28]. Hackmann et al. conducted research to produce 3D models of the canine stomach, demonstrating the potential of 3D scanning as an alternative tool for anatomical study [29]. The collected data can be used to generate highly accurate STL 3D models, which can be directly printed using 3D printers. Thomas et al. detailed the production of a frog skeleton model, capturing nearly all the details found in zoological textbooks. This procedure required minimal training and low costs with an initial investment in scanning equipment [30]. Similarly, scanning techniques introduce a more diverse range of species into the teaching process.

The aim of this study was to assess the effectiveness of using 3DP models in neuroanatomy course and to evaluate the level of student satisfaction with the use of these models. Based on the previous studies indicating positive results related to the use of 3DP models in education, it was hypothesized that:

- H1: 3DP models will improve students’ scores in veterinary anatomy assessment;
- H2: the use of 3DP models will increase students’ satisfaction with learning neuroanatomy;
- H3: the recognition of the anatomical structures in the colored 3DP models will be improved compared to the formalin-preserved specimens.

## Materials and methods

This study was approved by the Ethics committee of the University of Sarajevo, Bosnia and Herzegovina (0105-2853-1/24). Participation in the experiment was voluntary. Students were invited to participate and 90% of the total student group accepted and signed the consent. Informed consent was obtained from the 2022/2023 cohort specifically for two segments of the study: the assessment of students’ satisfaction with the 3D-printed models and participation in the recognition test.

### Specimen preparation

For this investigation, we utilized two brain models of the horse. The models were manually constructed from gypsum, based on images of a brain section. The first model illustrates the ventral surface of the brain, highlighting the origins of the cranial nerves, while the opposite side depicts a cross-section at the level of the lateral ventricles, mesencephalon, and rhomboid fossa. The second model represents a sagittal section of the brain, encompassing the cerebral hemisphere, cerebellum, and corpus callosum. The crucial aspect of these models was that they represent the most important parts of the brain in anatomical studies, as well as during necropsies, serving as representative samples for histological examination [31].

### 3D scanning of the brain model

For the 3D scanning of the brain models, the Artec Eva 3D scanner was used. This structured light 3D scanner enables creating quick, textured, and accurate 3D models of medium-sized and large objects. It is used for 3D scanning in both humans and animals, capturing precise measurements in high resolution rapidly. Utilizing safe structured-light scanning technology, it is capable of capturing objects of almost any kind, including those with black and shiny surfaces.

The 3D scanning and processing were performed using Artec Studio software. In the first step, all scans were cleaned, aligned together, registered using global registration and then STL 3D model was created. For engineering projects, this STL 3D model often needs to be imported into a CAD software to create a CAD 3D model. However, for medical or veterinary science applications, this step is simplified as the STL model is sufficient for future manufacturing using 3D printing (additive manufacturing).

**Fig. 1:**
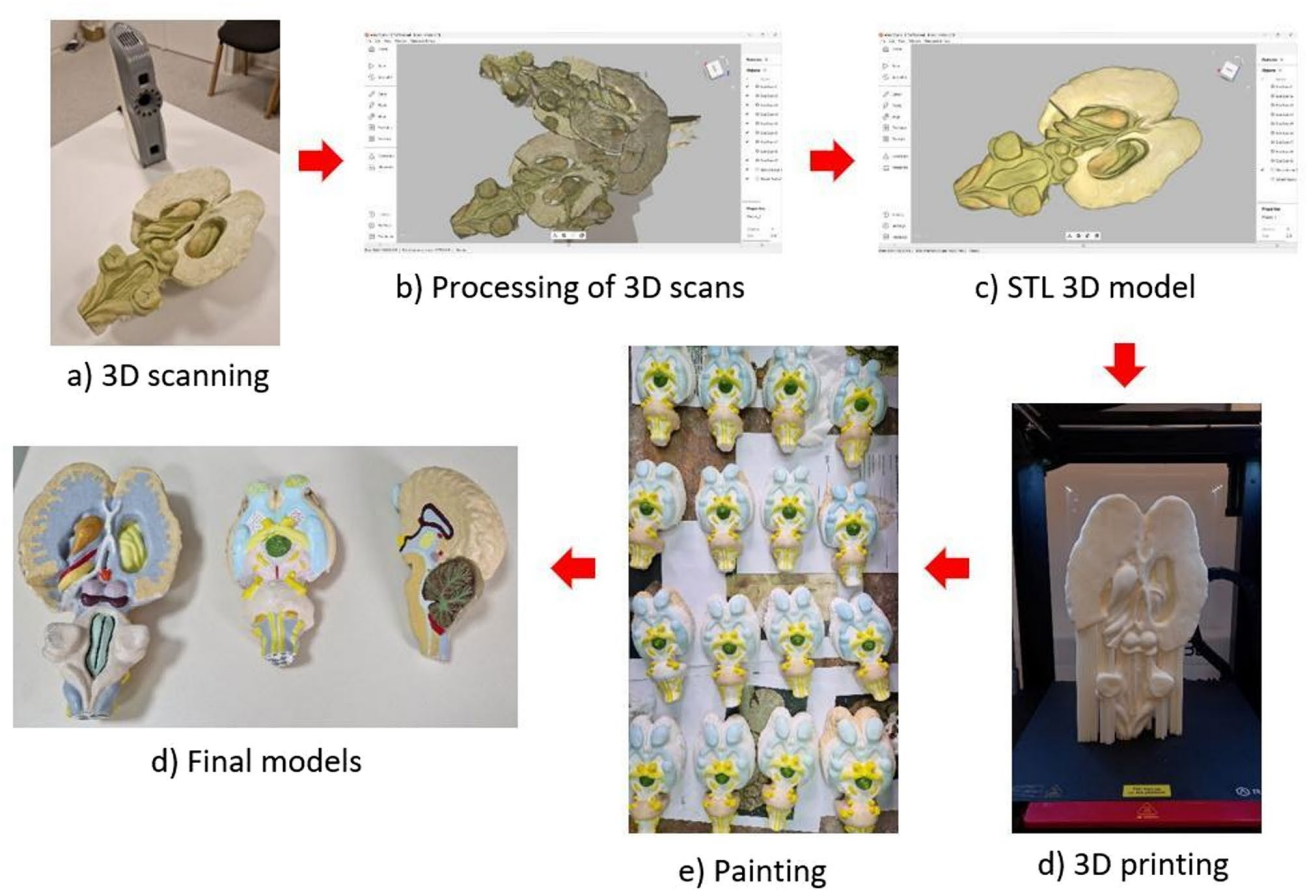
The flow chart of the process of preparing 3DP models

The first step in the process was to conduct the 3D scanning, making enough scans from different angles to capture the entire geometry of the brain model (Fig. 1a). The brain model can be moved and rotated between scans. For real organs, it is important that they remain in a solid form during the scanning process. The result of the 3D scanning is raw data comprising 9 scans. In the next step it is necessary to processes these scans, selecting those needed to construct the final model (Fig. 1b). The final step is to create a mesh STL model with the applied texture (Fig. 1c). The scanning software includes options for fixing errors that occur during processing or adding more scans if necessary. The STL model is then ready for manufacturing using 3D printing and can be further edited and smoothed if needed.

### 3D printing process

**Fig. 2:**
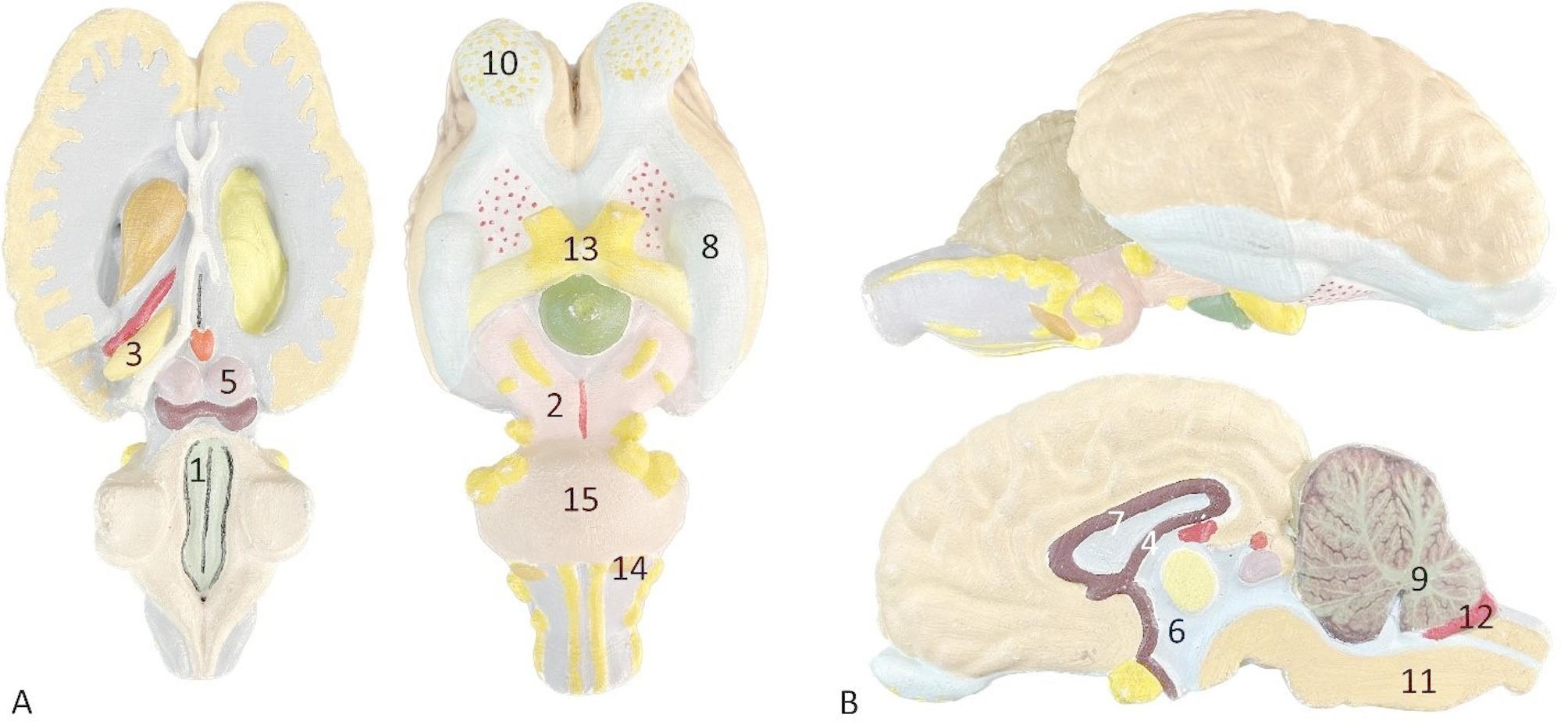
**A** Horizontal section of the brain with the ventral surface; (**B**) Sagittal section of the brain; 1, Fossa rhomboidea; 2, Pedunculi cerebri; 3, Cornu ammonis; 4, Fornix; 5, Corpora quadrigemina; 6, Ventriculus III diencephali; 7, Corpus callosum; 8, Lobus piriformis; 9, Arbor vitae; 10, Bulbus olfactorius; 11, Medulla oblongata; 12, Plexus chorioideus; 13, Chiasma opticum; 14, Corpus trapesoideum; 15, Pons cerebri

The 3D printing of the brain model was accomplished using FDM 3D printing technology, which involves extruding material (filament) through a preheated nozzle to create a product layer by layer on a print bed. Common materials for FDM technology include thermoplastics (PLA, ABS, Nylon, PC, PETG, etc.), though some 3D printers can also print composites and metals [32]. The STL model of the brain was prepared for 3D printing using slicer software Cura 5.6.0. The slicer for 3D printing converts a 3D model into a set of instructions (G-code) that a 3D printer uses for 3D printing. All 3D models were printed with predefined “Normal” printing parameters from the Cura slicer and in vertical printing orientation with a support structure using Ultimaker 3 and Raise3D Pro 3 Plus FDM 3D printers (Fig. 1d). The brain models were 3D printed using PLA (polylactic acid) material. Upon completing the printing process, the support structures were removed from all printed brain models. In the final phase, the 3D models were hand-painted with oil-paints, using different colors to indicate all anatomical structures for better recognizability (Fig. 1e). Anatomical validation of the models was conducted both during the preparation process and upon completion. A total of four professors from the Department of Anatomy were involved, and all steps were carried out under their direct supervision. This ensured the models’ morphological accuracy, dimensional fidelity, and structural integrity. A total of 32 models were prepared for practical sessions (Fig. 2).

### Study design

Experimental design is regarded as the most accurate method for researching the effects of innovations in teaching and education. However, because most educational interventions occur in natural settings, selecting a control or comparison group can be challenging. Control groups can be either concurrent or historical [33]. For this study, the experimental group consisted of the first-year students enrolled in the integrated study program at the University of Sarajevo-Veterinary Faculty during the summer semester of the academic year 2022/2023. Two historical control groups were chosen, whose results were obtained from the online data base of the Veterinary Anatomy course. Specifically, the experimental group’s data were compared to the results of the two previous student cohorts from the academic years 2020/2021 and 2021/2022, respectively. The disadvantages of historical to concurrent controls include the time distance between the groups, as well as differences in selection criteria and methods experienced by the two groups [34]. In our study, these disadvantages were minimized given the close temporal proximity of the three groups, all of which were taught by the same teacher. Additionally, all three cohorts included the first-year students and the enrolment criteria have remained consistent.

The neuroanatomy section comprises a total of 18 h over the course of three weeks. All students participating in the study took regular neuroanatomy classes, and were invited to participate in an experimental implementation of 3DP models in the teaching and learning process. The procedure did not account for students reporting color vision deficiencies (CVD), nor were any significant deviations observed in the recognition of anatomical structures that might indicate potential errors related to color recognition. Previous studies have indicated that medical professionals often remain unaware of their CVDs even after completing medical school and beginning their careers [34]. This yields the need for early screening for CVDs to develop systems solutions for both students and professionals [35]. However, in our research this did not show to be relevant.

The course was organized using 3DP models for 15 h, while during the remaining 3 h formalin-preserved specimens were utilized. This approach allowed students to compare and recognize anatomical structures on both types of specimens. A total of 32 3DP brain models were available, ensuring a 1:1 student-to-specimen ratio and facilitating an individualized learning experience, since students were divided into six groups in different time slots, but with the same teacher. Upon completing the three-weeks instruction, a neuroanatomy quiz was administered using the computer-aided learning management systems (LMSs) “Moodle”, which is regarded as an adequate platform for higher education [36].

Data obtained from this cohort were compared with the results of the previous two cohorts (2021–2022 and 2020–2021), who studied veterinary anatomy exclusively using formalin-preserved specimens. Consequently, the 2022/2023 cohort served as the experimental group, while the 2021–2022 and 2020–2021 cohorts served as comparative control groups. The average age of students was 19.8 ± 1.3 years.

The cohorts for the academic years 2020–2021 and 2021–2022 participated in practical sessions using formalin-preserved brain specimens from the Department of Anatomy’s Specimens Collection. There were 10 specimens available, with each one being shared by 2–3 students during the practical sessions.

To test the hypotheses, data was collected from three sources:

1. An online quiz was administered to evaluate students’ knowledge of anatomy across all three cohorts.
2. A paper-pencil questionnaire was distributed to assess students’ satisfaction with 3DP models, specifically for 2022–2023 cohort.
3. A recognition test was conducted on 3DP and formalin-preserved specimens to evaluate the recognition of the anatomical terminology. In this segment, only students for the 2022–2023 cohort were asked to identify 15 anatomical structures using both formalin-preserved and 3DP models (Fig. 2). Students answered orally and the teacher registered their answers in check lists prepared for each set of specimens.

Additionally, sex-based variations were scrutinized within each cohort and across the three of them. More than 400 questions were stored in the online question bank, and the system randomly selected 40 questions for a single quiz. The questions were prepared by a group of four teachers teaching Veterinary Anatomy course at the Veterinary Faculty. Three types of questions were included in the online quiz:

- Matching – students were given an image with anatomical structures labeled by numbers. Their task was to match each numbered structure with the correct anatomical term from a provided list of options;
- Multiple choice - allows the selection of one or more responses from a list;
- True/False - a simple dichotomous form offering only two choices.

**Fig. 3:**
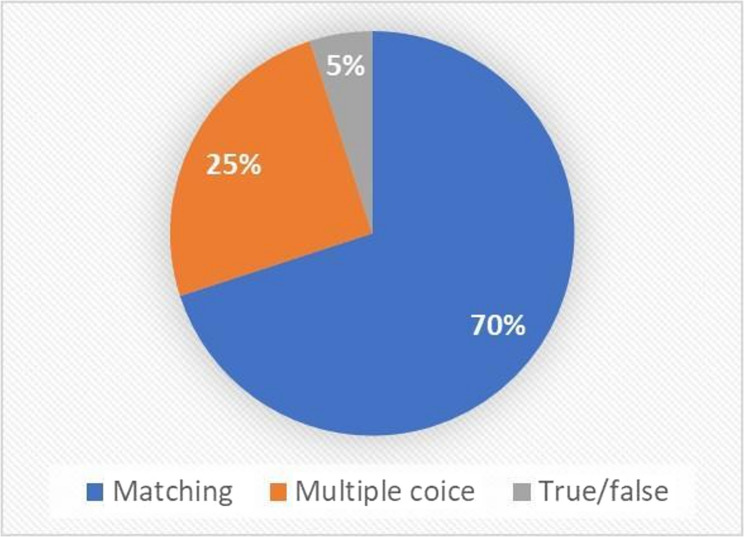
Question types in Moodle quiz

The dominant type in the quiz was matching questions, followed by multiple-choice (Fig. 3). The images used in the quiz were sourced from standard course literature and instructional materials, which were consistently available to all student cohorts.

Additionally, a questionnaire to assess students’ opinions about learning experience with the 3DP models compared to formalin-preserved ones in anatomy teaching was conveyed with the cohort 22/23. The questionnaire consisted of seven five-point Likert scale items and an open-ended item for additional comments about their learning experience with 3DP and formalin-preserved models (Table 3). In total, 25 students provided detailed elaborations of their experiences, which were then analyzed using the thematic analysis method [37, 38].

### Thematic analysis of students’ learning experiences with 3DP models

Data obtained from the responses to the open-ended question were analysed using thematic analysis to provide a deeper pedagogical understanding of 3D-printed models as a learning tool. The question asked: *What do you think about 3DP usage in Anatomy course?* By adding qualitative data, we tried to overcome reductionism that is present in numeric data, focusing only on assessments. Aligned with a social constructionist approach[39], this analysis rests on the premise that learners actively interpret and construct meaning from the educational tools and environments around them. In specimen-based learning, interaction between students and learning tools is especially relevant for the learning outcomes.

The process of thematic analysis followed the stages described by Braun and Clarke [37, 38]. It began with reading through the students’ responses while making initial notes about the data. This was followed by a systematic coding of the data, where recurring features were identified. These codes were then examined and grouped to develop initial themes. The emerging themes were reviewed and refined to ensure they accurately reflected the data. Each theme was then given a name. Finally, the findings were organized and presented in the results section. Two authors worked together during the process of thematic analysis and negotiated about the findings until reaching common conclusions in each of the stages. The final results were sent to the remaining seven authors for their review and their comments were also included in the final results.

### Recognition test

An additional evaluation of the use of 3DP models was conducting by comparing students’ recognition of neuroanatomical structures in both 3DP models and formalin-preserved specimens. The recognition test was designed to evaluate the educational value of the 3DP models, as indicated in the students’ performance scores based on identifying colored morphological structures. We selected the 15 most recognizable anatomical structures and asked students individually to identify these structures first using the 3DP models and then the formalin-preserved specimens. Students were exposed to both types of specimens simultaneously and, upon being asked a question, were required to indicate the anatomical structure on both specimens.

### Statistical analysis

The SPSS package program was used for statistical analysis (SPSS for Windows, version 22.0). To assess disparities in scores between groups and subgroups utilizing formalin-fixed anatomical specimens and those working with 3DP anatomical models, a one-way analysis of variance (ANOVA) was conducted. The ANOVA tested whether there were statistically significant differences in mean scores between multiple independent groups: males, females, and all students combined across the three cohorts (2020/21, 2021/22, and 2022/23). Prior to ANOVA, assumptions of normality and homogeneity of variance were checked using Shapiro–Wilk and Levene’s tests, respectively.

When the ANOVA yielded a statistically significant result (*p* < 0.05), a Tukey’s Honestly Significant Difference (HSD) post-hoc test was performed to determine which specific group means differed from each other while controlling for multiple comparisons. Tukey HSD is appropriate for balanced and unbalanced group sizes and is more conservative than unadjusted pairwise comparisons, reducing the likelihood of Type I error.

In addition to the ANOVA, independent-samples t-tests were conducted for selected pairwise subgroup comparisons to complement the post-hoc analysis. Subsequently, Hedges’ g test was applied for an analysis of the effect size [40]. Hedges’ g is preferred over Cohen’s d when sample sizes are small or unequal, as it includes a correction for bias. Interpretation followed conventional thresholds: small effect (g ≈ 0.20), medium effect (g ≈ 0.50), and large effect (g ≥ 0.80). Hedges’ g values were reported alongside t-test comparisons for transparency, regardless of statistical significance.

A paired-samples t-test was conducted to determine whether there was a statistically significant difference (*p* < 0.05) in the recognition of neuroanatomical structures between 3D-printed models and formalin-fixed specimens.

The Chi-square test was employed to assess disparities in the number of accurate and inaccurate responses following a learning period involving exposure to formalin-fixed anatomical specimens and 3D printed anatomical models. These statistical tests were instrumental in gauging the magnitude and statistical significance of the observed distinctions in performance across the various instructional methods.

## Results

### Students’ scores in veterinary anatomy assessment

In order to test the first hypothesis, the exam scores for the neuroanatomy course across the three cohorts included in the study were compared (Table 1). Passing exam scores ranged from 9 to 15 points.

**Table 1:** Analysis of average exam scores across three cohorts, stratified by sex

		cohort		
		2020/21	2021/22	2022/23
Average neuroanatomy exam score	Female	8.91	8.60	9.56
	Male	8.26	8.81	10.29
	Total	8.72	8.68	9.78

*Passing exam scores ranged from 9 to 15 points

A one-way ANOVA was conducted to assess differences in average student scores across 9 groups defined by cohort (2020/21, 2021/22, 2022/23) and gender (male, female, total). The results showed a statistically significant difference between groups, F(8, 397) = 2.07, *p* = 0.038. This suggests that at least one cohort-gender subgroup differed in performance. However, further analysis was required to determine which groups were responsible for the observed effect. To identify the specific group differences contributing to the significant ANOVA result, Tukey’s HSD post-hoc test was performed. Although the overall ANOVA showed statistical significance, Tukey’s HSD post-hoc comparisons did not reveal any individual pair of cohort-gender subgroups with a statistically significant difference after adjusting for multiple comparisons. This suggests that while score variability exists across cohorts and sex, no single contrast was strong enough to be statistically significant after correction. The largest mean difference was observed between male students in the 2020/21 and 2022/23 cohorts, although this difference did not meet the adjusted significance threshold (Tukey’s HSD *p* = 0.056).

To further explore subgroup trends, independent-sample t-tests were also conducted for selected pairwise comparisons. Several t-tests indicated statistically significant differences prior to correction (e.g., 2020/21 overall vs. 2022/23 overall: t(123) = 2.16, *p* = 0.032; Hedges’ g = 0.39). However, these did not remain significant after multiple testing corrections and are therefore considered exploratory in nature. Full results of both Tukey HSD post-hoc tests and independent t-tests with effect sizes are presented in Table 2.

**Table 2:** Exploratory and post-hoc comparisons of mean scores between student subgroups by cohort and sex, including uncorrected t-tests and Tukey HSD-adjusted *p*-values

Independent variables	*n*	Average exam score	SD	t	df	*p* (t-test)*	*p* (HSD)	Hedges’ g
All students - cohort 2020/21	63	8.72	3.03	0.07	142	0.93	0.99	0.01
All students - cohort 2021/22	81	8.68	2.73					
All students - cohort 2020/21	63	8.72	3.03	2.13	125	0.03	0.10	0.37
All students - cohort 2022/23	64	9.78	2.53					
All students - cohort 2021/22	81	8.68	2.73	2.47	143	0.01	0.05	0.41
All students - cohort 2022/23	64	9.78	2.53					
Male students - cohort 2020/21	18	8.26	2.7	0.76	61	0.44	0.97	0.21
Female students - cohort 2020/21	45	8.91	3.16					
Male students - cohort 2021/22	32	8.81	2.56	0.32	79	0.74	0.99	0.07
Female students - cohort 2021/22	49	8.6	2.86					
Male students - cohort 2022/23	19	10.29	2.05	1.04	62	0.30	0.72	0.28
Female students - cohort 2022/23	51	9.56	2.7					
Male students - cohort 2020/21	18	8.26	2.7	0.71	48	0.47	0.96	0.21
Male students - cohort 2021/22	32	8.81	2.56					
Male students - cohort 2020/21	18	8.26	2.7	2.58	35	0.01	0.12	0.84
Male students - cohort 2022/23	19	10.29	2.05					
Male students - cohort 2021/22	32	8.81	2.56	2.13	49	0.03	0.27	0.61
Male students - cohort 2022/23	19	10.29	2.05					
Female students - cohort 2020/21	45	8.91	3.16	0.49	92	0.62	0.93	0.10
Female students - cohort 2021/22	49	8.6	2.86					
Female students - cohort 2020/21	45	8.91	3.16	1.05	88	0.29	0.55	0.22
Female students - cohort 2022/23	45	9.56	2.7					
Female students - cohort 2021/22	49	8.6	2.86	1.67	92	0.09	0.15	0.34
Female students - cohort 2022/23	45	9.56	2.7					

*Given the absence of statistically significant results in the Tukey HSD post-hoc analysis, pairwise t-test comparisons presented here are unadjusted and should be interpreted as exploratory in nature

### Students’ satisfaction with learning neuroanatomy

As shown in Table 3, overall, students exhibit positive attitudes and report high satisfaction with using 3DP models in learning veterinary anatomy. They support innovative teaching methods and find that the colored segments of the models enhance recognizability of different anatomical structures, highlighting the didactical value of 3DP models. However, slightly lower ratings are observed when assessing the quality of the 3DP models and their adequacy as substitutes to formalin-preserved specimens, which opens the question for further examination of veracity of modeled specimens compared to the natural ones. Some indices, however, are found in students’ answers to the open-ended question. One student elaborated why she rated the item “3D printed models can be an effective substitute for formalin-preserved specimens” with a 2; *I think we should also have formalin-preserved specimens*,* because later*,* when we are in the situation of medical examination*,* we should know how the brain actually looks like inside* [F15]. Several other answers pinpoint that students find 3DP specimens valuable in education, but for real medical practice, formalin-preserved specimens are more valuable and accurate, as stated in a comment; *some structures are missing in 3DP specimens* [F14].

**Table 3:** Students’ satisfaction and attitudes to the use of 3DP models in veterinary anatomy (*n* = 50)

Statement	Mean ± SD
The anatomical features in the given 3D printed models are clearly visible	4.52 ± 0.57
The quality of the 3D printed models is satisfactory	4.38 ± 0.53
The colored parts of the model help me recognize different structures	4.76 ± 0.43
3D printed models enhance my understanding and learning of anatomy	4.46 ± 0.70
3D printed models can be an effective substitute for formalin-preserved specimens	3.90 ± 1.23
I feel safe using 3D printed specimens for learning	4.56 ± 0.61
Generally, I believe it is necessary to use innovative teaching methods	4.72 ± 0.57

*5 point Likert scale was utilized

In order to gain a deeper understanding of students’ experiences with using 3DP, answers to the open-ended question were analyzed using thematic analysis [41]. Students provided short and specific answers focusing on an actual aspect of 3DP specimens that they found important. In total, 25 student answers contained 30 lines for qualitative analysis. The second stage of the analysis process (initial data coding) led to eight codes being developed inductively, as presented in Table 4.)

**Table 4:** Thematic analysis codebook for the narrative responses about using 3DP

Code	Empirical description
3DP learning is more efficient	Describes how 3DP models help students better see, understand and memorise the specimen structure
It is easier to learn and understand anatomical structures by learning with 3DPs	How the physical 3DP models help students understand spatial relationships, orientation and scale
3DP makes it easier to recognise structures on formalin-prepared specimens	Ease of manipulation with the specimen
It is easier to work together in a student group using 3DPs	Describes how students work together to explain the anatomical structures while using the 3DP
Self-paced learning	Students can engage with the 3DP specimen at their own pace, even outside the classroom
Curiosity and wanting to learn more	It is more motivating to learn with innovative models
Reduced exposure to toxic substances	Relief due to avoiding contact with preserved biological specimens
Ethical and emotional comfort	3DPs reduce discomfort or ethical concerns associated with using biological specimens

Further defining of the themes was based on the questions proposed by Braun and Clarke[42] asking about is it a theme or it could be just a code, what is the quality of the theme, what are its boundaries, are there enough data to support a particular theme or are the data diverse or is there a coherence in the theme. This resulted in naming three recurring themes reflecting advantages of 3DP specimens over the formalin-preserved ones; (1) better clarity of the specimen, (2) better interaction between students and the specimen, (3) and health safety (Table 5).

**Table 5:** Empirical description of the main themes found in the thematic analysis

Theme	Acronym	Empirical description	Examples from the data
Specimen clarity	SPC	Includes students’ views on how visible are structures and elements on the 3D printed specimens	Better visibility and recognition of the structures, facilitated learning process, enhances understanding of complex structures
Student-specimen interaction	SSI	Describes how students manipulate with the 3D printed specimens	Better navigation and connectivity of the study content, using different colors and labels, easy-to-use
Health safety	HES	Describes whether 3D printed specimen causes any health issues	Neutral smell, material is not harmful for chronical respiratory patients

In students’ elaborations, clarity of the 3DP specimens is particularly associated with coloring; *with different colors used to label different parts*,* I can easily distinguish and identify them.* [F02]

*The 3D printed model was a great help to me because I found it easier to recognize structures compared to formalin preparation. [F03]*

 The interaction between students and specimens was enhanced with 3DP models given their firmness and the possibility of producing the necessary number of models according to the students’ needs. Health safety is an important aspect that arose from the qualitative content analysis and was not considered prior to initiating the study. Students with chronic respiratory difficulties and allergies reported that they felt much safer with 3DP given that in formalin specimens *the “odor” is terrible causing serious health issues* [M07].

Innovation in teaching and learning neuroanatomy was in itself perceived positively by students, who particularly saw better didactical value in 3DP specimens. Therefore, students’ attitudes and satisfaction about these types of specimens was high and positive leading to confirmation of the second hypothesis.

### The colored 3DP specimens and effects on recognition

The Chi-square test revealed a significant difference, χ2 (1, *N* = 1110) = 3.99, *p* = 0.0455) in the number of accurate and inaccurate responses following a learning period involving practice with formalin-fixed anatomical specimens and 3D printed anatomical models across three cohorts, as indicated in Table 6. The 15 most recognizable anatomical structures were selected for this purpose according to Fig. 2.

**Table 6:** Comparison of correct and incorrect responses after practice period with formalin-fixed anatomical specimens and 3D printed anatomical models over three years of examinations

	Practicing with formalin-fixed anatomical specimens	Practicing with 3D printed anatomical models
Correct answers	438	464
Incorrect answers	117	91

**Fig. 4:**
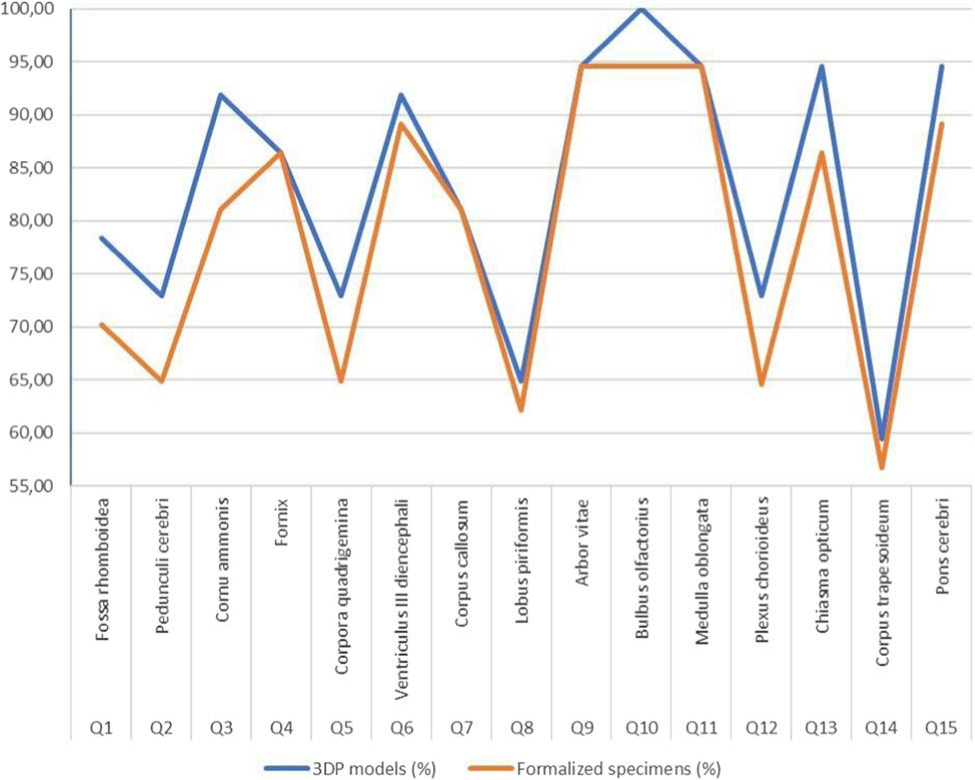
Comparing students’ recognition of the neuroanatomical structures in the 3DP models and formalin-preserved specimens, *n* = 37

Based on the recognition test presented to the 2022/23 cohort, it can be seen that the ratio of correct answers in the 3DP specimens ranges from 59.45 to 100%. In formalin-preserved specimens, the correct answers ranged from 56.75 to 94.59%. Students performed significantly better when identifying anatomical structures on 3DP models compared to formalin-preserved specimens. A paired-samples t-test demonstrated a statistically significant difference in the identification of anatomical structures between 3DP and formalin-preserved specimens (t(14) = 4.80, *p* = 0.00023), with higher accuracy observed when using the 3DP models (Fig. 4).

## Discussion

Visuo-spatial tools facilitate a comprehensive understanding of complex three-dimensional anatomical structures. The application of 3D scanning technology has proven highly effective not only in educational contexts but also as a valuable asset in research. The exceptional precision and accuracy afforded by this technology are crucial for conducting detailed geometric studies in three dimensions [43, 44].

The primary result, focused on neuroanatomy test scores, demonstrated a significant improvement for the 22/23 cohort. This enhancement was predominantly attributed to male students, who achieved statistically significant higher scores when utilizing 3DP models compared to the preceding two cohorts that utilized formalin-fixed specimens. Gender differences in visuospatial skills reveal that males score higher than females, particularly on tests involving three-dimensional mental rotation tasks [45, 46]. This study demonstrated that 3DP models were highly beneficial for neuroanatomy learning, pedagogically useful and well-received by students. Several randomized controlled studies have demonstrated the positive impact of utilizing 3DP models for medical and dental students. Specifically, students who engaged with 3DP skulls and pelvic lymph node models achieved higher test scores than those in the control group, who relied on traditional cadaveric skulls and anatomical atlases [47–49]. In a study by Wu et al. the effectiveness of 3DP models was compared to radiographic images for teaching spatial anatomy and fractures, with findings indicating significantly longer test-taking times for the traditional radiographic group [50]. The important benefit of using 3DP models for veterinary clinical skill training was the ability to ease the transition to working with live animals by reducing stress [51].

Positive attitudes and high levels of student satisfaction were reported with the use of 3DP models. However, the lowest satisfaction scores were associated with the replacement of formalin-preserved specimens by printed models. First-year anatomy students perceived real tissue specimens as crucial for comprehensive learning, feeling that the absence of real tissue and cadavers will lead to an incomplete educational experience. Similar findings were noted in the study by Diaz-Reganon et al. which examined the use of upper endoscopy models for both anatomy learning and clinical simulation [52]. Conversely, a significant concern highlighted by students was the health risk associated with working with formalin-preserved specimens. Despite preparation methods aimed at reducing formalin concentration, students continued to perceive a strong odor and expressed health-related worries. This feedback underscores the importance of minimizing the use of formalin-preserved specimens where feasible to ensure a safer learning environment.

The highest overall student satisfaction score was achieved for the color-coding of different anatomical structures on brain models, with a mean rating of 4.76 ± 0.43. This feature was highly recommended as it proved exceptionally beneficial for identifying and distinguishing various brain regions. A similar finding was observed in the teaching of upper limb anatomy [27]. Satisfaction with color-coding was strongly correlated with students’ performance on recognition tests (Table 6). In four out of fifteen anatomical structures, students’ responses were consistent for both types of specimens, while for the remaining structures, students demonstrated higher accuracy when using the 3DP models.

The accuracy of 3DP models is essential for supporting effective learning outcomes. Neuroanatomical models, in particular, are well-suited for such studies due to the intricate and precise structures that must be identified. Additionally, 3D printing offers significant advantages in neuroanatomy education, including cost-effectiveness, ease of use, and durable materials, making it a practical and sustainable option [53]. The initial investment in a 3D printer typically ranges from $500 to $3,000 USD. The material cost for producing a single brain model is under $5 USD, with an additional similar amount for painting services.

### Limitations

The limitations of this study included the comparison of results with only two historical cohorts. The later cohort was excluded from consideration due to the impact of the COVID-19 pandemic and the specific educational and assessment conditions during the spring of 2020. Additionally, a potential Limitation could be the number of models used, as the study focused on two of the most important sections prepared specifically for this purpose. Incorporating additional models representing different sections would provide significant benefits for students, enhancing their understanding and learning experience. The use of 3D scanning and printing technology is essential in this process, as it facilitates the creation of a diverse range of models, either derived from real anatomical specimens or based on manually prepared prototypes. Another potential Limitation is that the evaluation of the 3DP models was conducted exclusively by students. In future studies, it will be important for 3DP models to also be assessed by anatomy educators and practitioners to provide a more comprehensive evaluation.

## Conclusions

This study explored the effectiveness of integrating 3DP models into the neuroanatomy curriculum. It evaluated students’ test scores, satisfaction levels, and recognition performance by comparing the use of 3DP models with traditional formalin-fixed specimens. Students achieved higher scores when using the 3DP models. Key advantages of the models, as identified by the students, included color coding, health safety, and the ease of recognizing anatomical structures. These findings indicate that 3DP models offer significant benefits for both anatomy students and educators. Integrating 3D technology into the anatomy curriculum could enhance learning outcomes and expand instructional possibilities by supplementing formalin-fixed specimens with highly detailed, reusable 3DP models.

## Data Availability

The data that support the findings of this study are available from the corresponding author upon reasonable request. The 3D models used in this study are accessible on the official Sketchfab profile of the University of Sarajevo – Veterinary Faculty, available at this link [https://skfb.ly/psoOD]. The 3D print files can be provided free of charge upon request.
